# Substance use in cancer patients – an undervalued and harmful association

**DOI:** 10.1192/j.eurpsy.2025.1789

**Published:** 2025-08-26

**Authors:** A. L. Ramos, J. T. Coelho, H. Salgado

**Affiliations:** 1 São João Local Health Unit; 2 Faculty of Medicine of University of Porto, Porto, Portugal

## Abstract

**Introduction:**

Substance use and substance use disorders are widespread among cancer patients, throughout the complex course of the disease. Besides the fact that the use of some substances predisposes individuals to cancer, substance use can also develop after a cancer diagnosis. The latter becomes more concerning by the significant use of opioids in the context of cancer pain management. In addition, individuals with cancer may use substances in an attempt to cope with psychological distress or poorly controlled physical symptoms – “chemical coping”.

**Objectives:**

In this work, we intend to do a review on the prevalence and impact of substance abuse in oncological population.

**Methods:**

Our research will focus on papers of the last 10 year, indexed in Pubmed. We will summarize the most relevant data for clinical practice.

**Results:**

The use of substances in cancer patients is extremely heterogeneous. Not all of the situations constitute a Substance Use Disorder categorized in the diagnostic manuals used in psychiatry. Nevertheless, substance abuse always represents a causal agent in the deterioration of clinical and functional state of cancer patients.

According to studies, cancer patient care services do not assess patients substance abuse patterns or risk in an assertive and systematic manner. Moreover, there are no specific guidelines for the approach and treatment of cancer patients with addictive disorders.

Some authors also emphasize that the stigma associated with patients with substance dependences can difficult their access to health care and delay the diagnosis of neoplastic diseases or prevent them from undergoing treatment, which is often prolonged.

**Conclusions:**

As a conclusion, we highlight that further studies are needed in this population, with more attention to the interaction between substance use and worse clinical progression. Furthermore, we remind the increasingly need for multidisciplinary teams to approach these patients - from oncology to psychiatry. There should be a greater investment in training these teams, as it can significantly improve the treatment and prognosis of these patients.

**Disclosure of Interest:**

None Declared

